# Early diagnosis and aggressive intervention in *Vibrio vulnificus* infection following live fish dorsal fin puncture: a case report

**DOI:** 10.3389/fmed.2025.1593819

**Published:** 2025-07-29

**Authors:** Erxiang Zhang, Zhen Chen, Du Feng, Gengfeng Chen, Delong Yin

**Affiliations:** ^1^Department of Orthopedics, The Third Affiliated Hospital, Guangzhou Medical University, Guangzhou, China; ^2^Guangdong Provincial Key Laboratory of Major Obstetric Diseases, Guangdong Provincial Clinical Research Center for Obstetrics and Gynecology, The Third Affiliated Hospital, Guangzhou Medical University, Guangzhou, China; ^3^Nanshan School of Medical, Guangzhou Medical University, Guangzhou, China; ^4^First Clinical School of Medical, Guangzhou Medical University, Guangzhou, China

**Keywords:** *Vibrio vulnificus*, stab wound, wound infections, early intervention, fish

## Abstract

In this paper, we report a case of traumatic wound infection caused by dorsal fin puncture of live fish. A 69-year-old woman developed progressive swelling of her right pinky finger after being stabbed by the dorsal fin of a live fish. The infection was confirmed by bacterial culture as a *Vibrio vulnificus* infection of a traumatic wound. The patient underwent antibiotic treatment, surgical decompression, debridement, and excision of necrotic tissue. Finally, the right pinkie finger was amputated due to dry gangrene. Early intervention and combined antibiotic therapy led to a good prognosis. As global ocean temperatures rise, the infection rate of this bacterium increases, and vigilance is needed. Clinical practice suggests that such infections should be considered in patients with a history of contact with seafood or seawater; Early diagnosis, active antibiotic therapy and necessary surgical intervention are the keys to improving the prognosis.

## Introduction

*Vibrio vulnificus* is a Gram-negative, halophilic bacterium that is widely distributed in warm marine environments. It is an opportunistic pathogen that usually causes wound infections and gastroenteritis through contact with contaminated seawater or consumption of contaminated seafood, and in severe cases can be life-threatening due to sepsis. The elderly and individuals with underlying diseases such as liver disease, diabetes, and immune deficiency are at high risk for *Vibrio vulnificus* infection. As global warming causes ocean temperatures to rise, *Vibrio vulnificus* infection rates are on the rise, making it the food-borne pathogen with the highest case fatality rate (1, 2). In this case report, we report a case of *Vibrio vulnificus* infection that necessitated early amputation due to a live fish’s dorsal fin injury to the finger.

## Case presentation

**Figure 1:**
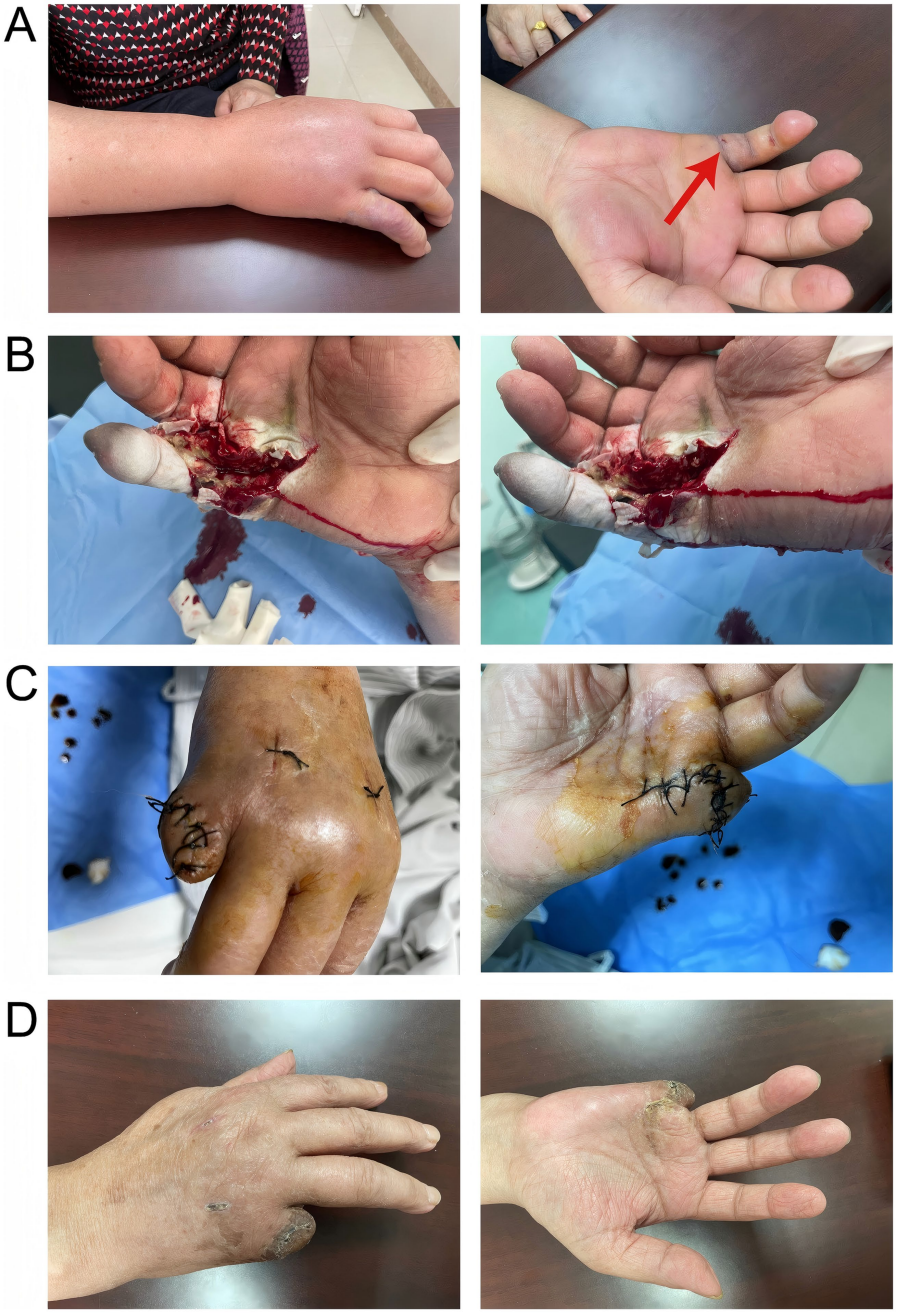
**A** represented the infection condition of the patient’s right hand and forearm at the time of consultation; **B** showed the surgical process of incision, decompression, and drainage; **C** depicted the amputation of the little finger of the right hand and suturing of the stump on March 31, 2023; **D** illustrated the well-healed wound with no signs of infection.

**Figure 2:**
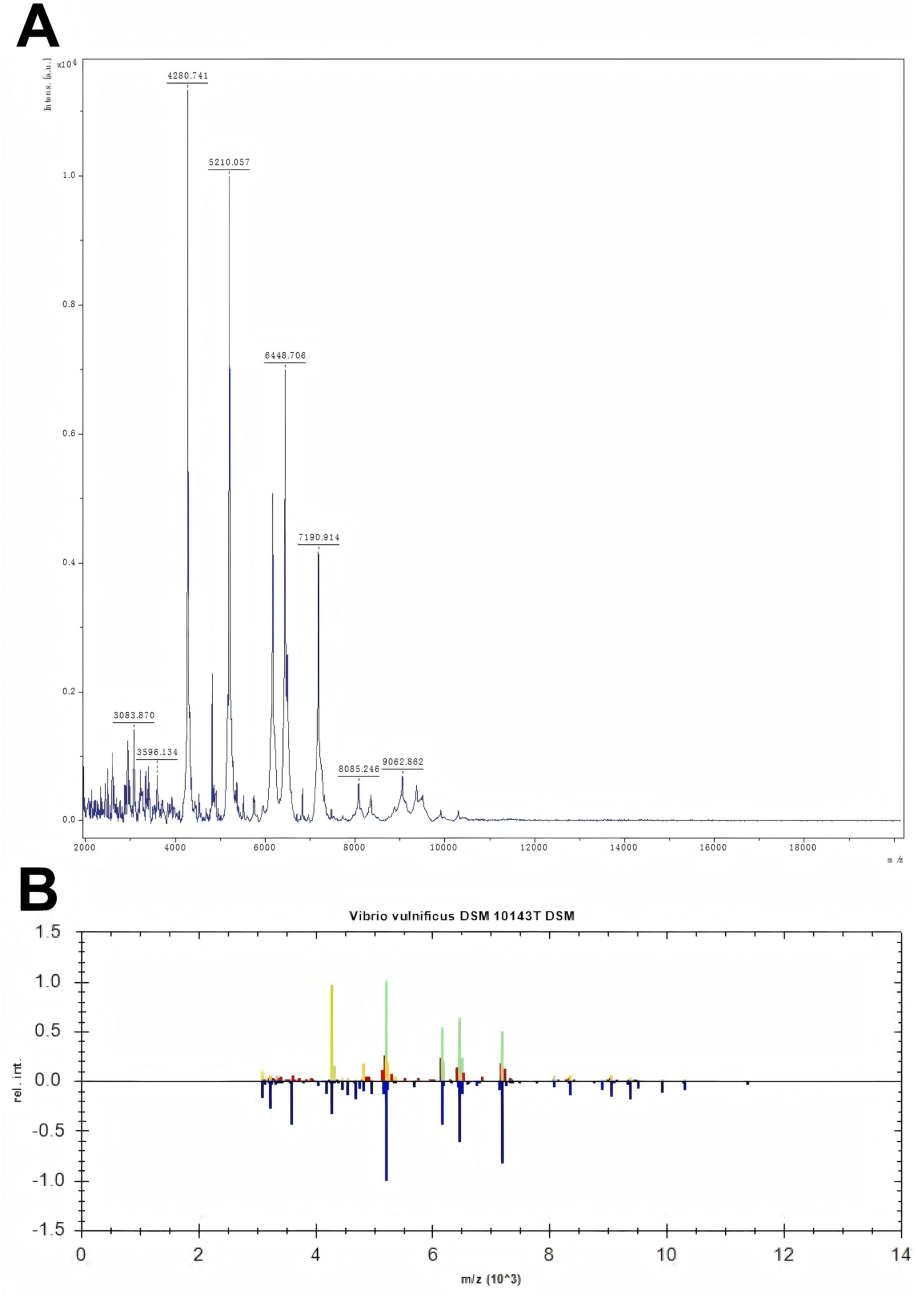
Figure **A** shows the mass spectrometry identification peak chart of the detected bacteria. In the mass spectrum, the X-axis represents the mass-to-charge ratio (m/z), which is the ratio of mass to charge, and the Y-axis represents the relative intensity. Figure **B** shows the comparison of the peak charts between the detected bacteria and the standard bacteria. The upper part is the peak chart of the detected bacteria, and the lower part is the peak chart of the standard bacteria from the bacterial library. By comparing the two, green indicates that the two have the same peaks. The more green appears, the more it suggests that the detected bacteria are *Vibrio vulnificus*.

A 69-year-old woman with a 3-year history of hypertension was stabbed by the dorsal fin of a live fish on her right little finger on March 4, 2023, and presented with persistent pain and progressive enlargement of her right hand. The patient came to the clinic on March 6, showing swelling of the right hand and right forearm, limited movement, elevated local skin temperature, obvious swelling of the right little finger with ecchymosis, fluctuation, and a stab wound at the palmar root (Figure 1A). Unfortunately, because the patient did not know what kind of fish stabbed her, we did not know the species of fish. Laboratory tests showed that at admission, the white blood cell count was 30.26 × 10^9/L, the neutrophil percentage was 91.80%, the rapid C-reactive protein (CRP) level was 200.00 mg/L, the procalcitonin level was 9.07 ng/mL, the serum creatinine (Scr) level was 85umol/L, the serum potassium level was 2.96 mmol/L, and the uric acid content was 362umol/L. Other tests, such as liver function, chest film, electrocardiogram, did not show significant abnormalities. The preliminary diagnosis was an infection caused by a dorsal fin puncture of a live fish on the little finger of the right hand, and the patient was admitted for treatment. Starting on March 7, 2023, the patient was administered vancomycin 500 mg intravenously every 12 h, and cefotaxime sodium with sulbactam sodium (2:1) 1.5 g intravenously twice daily. Considering the local swelling and limited mobility of the patient’s right hand, on March 8, 2023, fasciotomy and decompression of the right hand with Vacuum Sealing Drainage were performed to provide incision and drainage, thereby preventing the infection from spreading throughout the body. Tissue samples were taken for pathogen identification. Based on the matrix-assisted laser desorption/ionization time-of-flight mass spectrometry (MALDI-TOF MS) technology, the protein fingerprint patterns of bacteria were detected. The specific method employed was as follows: After mixing the bacterial samples with the matrix and ionizing them, charged peptide segments and protein fragments were formed. Characteristic mass spectrometry peaks were generated based on the differences in migration time of their mass-to-charge ratio (m/z) in the flight tube (Figure 2A). By comparing these results with the reference atlas of *Vibrio vulnificus* standard strain database (Figure 2B), matching the key peaks, and calculating the similarity score, it was confirmed that the infection was caused by *Vibrio vulnificus*. Because the patient’s condition was urgent, we did not perform culture on TCBS (thiosulfate–citrate–bile salts–sucrose) agar or other similar selective medium. However, we conducted a drug resistance test on *Vibrio vulnificus*, and the results showed that it was sensitive to 12 kinds of antibiotics such as ceftazidime, cefoperazone, and levofloxacin. On March 13, 2023, the anti-infection regimen was changed to intravenous infusion of cefoperazone sodium and tazobactam sodium (8:1) 2.25 g every 12 h, and intravenous infusion of levofloxacin 0.5 g once daily. On March 17, 2023, the anti-infection regimen was changed to intravenous infusion of cefoperazone sodium and sulbactam sodium (1:1) 1.5 g twice daily, and intravenous infusion of levofloxacin 0.5 g once daily, which was continued until April 3, 2023.

The patient underwent a local excision or destruction of the diseased or other local tissues of the skin and subcutaneous tissue of the right hand on March 16, 2023, and March 24, 2023, respectively, for further drainage and debridement (Figure 1B). During the postoperative rounds on March 24, 2023, it was found that the little finger of the patient’s right hand was still black and shriveled, with reduced sensation and lower skin temperature, which was considered dry gangrene of the right little finger. Subsequently, on March 31, 2023, the patient underwent amputation of the little finger of the right hand and skin repair of the stump (Figure 1C). Throughout the nearly one-month treatment period, symptomatic treatments such as anti-infection, anti-edema, and analgesia were continuously provided. Through early intervention, the symptoms of *Vibrio vulnificus* infection in the patient’s right hand were gradually alleviated, and the wound of the little finger stump healed well (Figure 1D). On April 4, 2023, the patient was discharged with instructions to take cefdinir capsules three times daily, 0.1 g each time, for a total of 7 days.

## Discussion

*Vibrio vulnificus* is a zoonotic, Gram-negative bacterium known to cause septicemia and wound infections in humans (3). In some reported cases, infections arise through the consumption of inadequately cooked or contaminated seafood, especially bivalve shellfish such as oysters, clams, and mussels (1, 4). Additionally, improper storage or transportation at sub-optimal temperatures (e.g., failure to maintain the cold chain), contamination by infected food handlers, or direct contact with contaminated seafood or seawater can result in infection (5). Although *Vibrio vulnificus* infection is relatively rare, with approximately 100 documented infections annually in the United States, it poses significant health risks to immunocompromised or susceptible individuals. Moreover, global ocean warming has substantially increased the geographical regions favorable for this pathogen’s proliferation, thereby contributing to heightened public health concerns and posing additional challenges to current prevention and surveillance measures (2).

*Vibrio vulnificus* infection is broadly categorized into three principal forms: primary septicemia (43.1%), traumatic infections (45.9%), and gastroenteritis typically linked to raw seafood intake (5%) (6). Infections unfold swiftly, with wound-related cases averaging a 16-h incubation period and septic events occurring around 26 h post-exposure (7). Common gastrointestinal manifestations include constipation, nausea, and vomiting (8). For wound-associated *Vibrio vulnificus* infections, a 17% mortality rate has been documented, frequently presenting as cellulitis, petechiae, or maculopapular lesions that can progress to necrotizing fasciitis (9). Primary septicemia, the most lethal clinical form, carries mortality rates surpassing 50% and is characterized by fever, chills, digestive upset, hypotensive shock, and secondary lesions on the extremities (9). In the patient described here, a dorsal fin puncture on the right little finger and subsequent two-day progression to hand and forearm swelling signified a traumatic infection rather than sepsis. Notably, the absence of septic manifestations may reflect the patient’s favorable physical condition, the lack of major comorbidities, and rapid initiation of antibiotic treatment. Previous research indicates that *Vibrio vulnificus* exhibits a marked predilection for males (approximately 90%) and the older adults (about 85% of cases involve individuals beyond 40 years). High-risk populations commonly include those with underlying conditions such as chronic liver dysfunction, diabetes mellitus, hemochromatosis, AIDS, malignancies, or compromised immune status (10). In this particular case, the reason why primary sepsis did not occur may be related to the fact that the patient did not have many relevant risk factors, was in better physical condition, and received timely antibiotic therapy based on drug sensitivity test results.

Infections triggered by *Vibrio vulnificus* frequently lead to life-threatening outcomes, with patient prognosis tightly associated with swift and precise diagnosis alongside appropriate treatment. While diagnosing *Vibrio vulnificus* remains comparatively straightforward, the core laboratory approach involves culturing blood, fecal, or wound secretion samples, recognized as the gold standard for confirming clinical infection (8). Biochemical evaluations may reveal elevated white blood cell counts, marked platelet reductions, coagulation abnormalities, metabolic acidosis, and hepatic or renal impairment (11, 12). Due to unforeseen conditions that we did not cultivate the pathogen in TCBS medium at that time. However, based on reasonable assumptions, we believe that *Vibrio vulnificus* presents a green colony on the TCBS medium (13–15). These colonies are round in shape, with a smooth surface, neat edges and a semi-transparent texture. Initial drug-susceptibility findings suggest *Vibrio vulnificus* responds to a range of antimicrobial agents. Following official recommendations from the United States Centers for Disease Control and Prevention (CDC), a combination therapy consisting of a third-generation cephalosporin and tetracycline is considered first-line for *Vibrio vulnificus* management (16). Notably, some research posits that quinolones may exhibit superior efficacy relative to tetracyclines, whereas cephalosporins can deliver enhanced results when paired with quinolones (17, 18). In this case, the patient rapidly developed local inflammation and fingertip blackening after a stab wound. Early surgery failed to relieve symptoms, suggesting possible acute Gram-positive infection (19). Vancomycin-used for severe Gram-positive infections, especially Methicillin-resistant *staphylococcus aureus* (MRSA) was considered (20). Based on Mohammedi’s recommendation of a 15 mg/kg loading dose and input from infectious disease specialists, empirical vancomycin therapy was started to prevent worsening (21). We used the third-generation cephalosporin (a broad-spectrum antibiotic) targeting Gram-negative bacteria and vancomycin targeting Gram-positive bacteria on the patient in the early stage. This drug combination encompasses Gram-positive bacteria and Gram-negative bacteria very well. A prompt revision of the therapeutic strategy following definitive diagnosis, employing third-generation cephalosporins alongside quinolones, demonstrated enhanced effectiveness in arresting the infection. In combination with routine surgical incision and drainage, these measures curbed further spread and prevented systemic sepsis. It was observed that diverse antibiotic regimens did not significantly alter patient prognoses, whereas delayed surgical intervention correlated with elevated risks of deterioration and fatality (22). Timely debridement, as well as necessary amputation, remains pivotal for improving outcomes in *Vibrio vulnificus* infection (23). Under local or intravenous analgesia, the affected limb undergoes incision through skin and subcutaneous tissue, bluntly separating layers to expose fascia and muscularis propria. Incision techniques were determined by coagulation status, skin appearance, and lesion size. Postoperatively, gauze saturated with iodophor and sulfadimethoxine solution was externally applied to promote dressing changes and expedite wound assessment. Vigorous debridement, targeted dermatoplasty, and comprehensive supportive treatment can improve patient outcomes (24). Despite confirming the absence of *Vibrio vulnificus* in follow-up cultures, the patient nevertheless experienced unavoidable gangrene during subsequent care. Elevated iron levels in infected individuals show a strong correlation with *Vibrio vulnificus* pathology (25). This dynamic may relate to haemolysins and extracellular proteases VvpE, as the organism potentially capitalizes on haemolysin activity to secure iron, thereby intensifying disease severity. Additional haemolysin-associated manifestations include fluid accumulation, intestinal disturbances, partial paralysis, and potential fatality. Exposure to toxins substantially increases vascular permeability, initiates endothelial cell apoptosis, elevates inducible nitric oxide synthase function, amplifies nitric oxide production, and may spur neutrophil migration, culminating in maculopapular lesions (7). These mechanisms underpin the patient’s initial clinical manifestations, in conjunction with *Vibrio vulnificus* endotoxin, which can affect clinical outcome once bacterial cells are lysed (26). To halt necrotic spread, we excised the little finger. According to the International Federation of Societies for Surgery of the Hand (IFSSH), the little finger accounts for only 9–10% of hand functionality, validating the minimal influence on overall hand performance when excised. Prompt intervention restricted the extent of amputation to a single digit, thereby reducing impact on fundamental hand function.

## Conclusion

We reported a case of *Vibrio vulnificus* infection caused by a live fish dorsal fin injury to the finger. The patient had a good prognosis due to early and aggressive surgical intervention combined with antibiotic therapy. *Vibrio vulnificus* infection should be considered in individuals who have developed skin infections with a history of exposure to contaminated seafood or seawater. Early and aggressive antibiotic therapy, supportive care, and surgery are critical for improving outcomes and saving patients’ lives.

## Data Availability

The data that support the findings of this study are available on request from the corresponding author, Delong Yin, upon reasonable request.
